# Pevonedistat, a NEDD8‐activating enzyme inhibitor, induces apoptosis and augments efficacy of chemotherapy and small molecule inhibitors in pre‐clinical models of diffuse large B‐cell lymphoma

**DOI:** 10.1002/jha2.2

**Published:** 2020-04-09

**Authors:** Pallawi Torka, Cory Mavis, Shalin Kothari, Sarah Belliotti, Juan Gu, Suchitra Sundaram, Matthew Barth, Francisco J. Hernandez‐Ilizaliturri

**Affiliations:** ^1^ Roswell Park Comprehensive Cancer Center Buffalo New York USA; ^2^ Yale University School of Medicine New Haven Connecticut USA

**Keywords:** Apoptosis, MLN4924, NF‐κB, non‐Hodgkin lymphoma, ubiquitin proteasome system (UPS)

## Abstract

We studied the biological activity of pevonedistat, a first‐in‐class NEDD8‐activating enzyme (NAE) inhibitor, in combination with various cytotoxic chemotherapy agents and small molecule inhibitors in lymphoma preclinical models. Pevonedistat induced cell death in activated B‐cell (ABC) diffuse large B‐cell lymphoma (DLBCL) cell lines and to a lesser degree in germinal center B‐cell (GCB) DLBCL cell lines. In pevonedistat sensitive cells, we observed inhibition of NF‐κB activity by p65 co‐localization studies, decreased expression of BCL‐2/Bcl‐XL, and upregulation of BAK levels. Pevonedistat enhanced the activity of cytarabine, cisplatin, doxorubicin, and etoposide in ABC‐, but not in the GCB‐DLBCL cell lines. It also exhibited synergy with ibrutinib, selinexor, venetoclax, and A‐1331852 (a novel BCL‐XL inhibitor). *In vivo*, the combination of pevonedistat and ibrutinib or pevonedistat and cytarabine prolonged survival in SCID mice xenograft models when compared with monotherapy controls. Our data suggest that targeting the neddylation pathway in DLBCL is a viable therapeutic strategy and support further clinical studies of pevonedistat as a single agent or in combination with chemotherapy or novel targeted agents.

## 1 INTRODUCTION

Diffuse large B‐cell lymphoma (DLBCL) represents the most common subtype of non‐Hodgkin lymphoma (NHL), comprising 30–40% of all newly diagnosed cases [1]. The 2016 WHO classification recognizes germinal center B‐cell type (GCB‐DLBCL) and activated B‐cell type (ABC‐DLBCL) as molecular subgroups of DLBCL [2, 3]. ABC‐DLBCL is associated with constitutive activation of the nuclear factor kappa B (NF‐κB) pathway [4] that leads to chemotherapy resistance and inferior clinical outcomes. Multistep targeting of the NF‐κB pathway using investigational and currently available small‐molecule inhibitors could result in novel, active, and potentially less toxic regimens for ABC‐DLBCL patients.

The ubiquitin proteasome system (UPS) regulates BCL‐2 family members and hence the apoptotic cascade, either indirectly by altering function of the NF‐κB pathway (leading to an increase of BCL‐2, MCL‐1, and BCL‐XL levels) or by degrading pro‐apoptotic BCL‐2‐related proteins (such as BAK) [5, 6]. Inhibition of the UPS is an attractive therapeutic approach in lymphoid malignancies, including DLBCL. Proteasome inhibitors have been used in the treatment of lymphoma; however, their use is limited due to adverse effects [7, 8].

Proteasomal degradation of cellular proteins is a multistep process that requires “tagging” of targeted proteins with poly‐ubiquitin chains. The ubiquitin ligases (E3s) are responsible for selectively recognizing substrates [9] and their diversity presents an attractive target for more selective inhibition of the UPS that may potentially be more clinically effective and less toxic. Activation of cullin‐based RING‐ubiquitin ligase (CRLs) requires neddylation of the cullin subunit, which disrupts its inhibitory binding to the cullin‐associated NEDD8‐dissociated protein 1 (CAND1) [10]. Neddylation is a posttranslational modification that involves the addition of the ubiquitin‐like protein, NEDD8, to a target protein (E3s). Pevonedistat is a first‐in‐class, NEDD8‐activating‐enzyme (NAE) inhibitor that selectively prevents the activation of CRLs and alters the ubiquitination and proteasomal degradation of cellular proteins. Inhibition of NAE led to cell death in various cancer models [11, 12, 13, 14]. *In vitro* exposure of cancer cell lines to pevonedistat was shown to induce apoptosis, cellular senescence, or autophagy [11, 14, 15, 16]. We characterized the preclinical activity of pevonedistat as a single agent and in combination with chemotherapy and other novel agents in DLBCL.

## 2 MATERIALS AND METHODS

### 2.1 Cell lines and primary tumor cells

RL (GCB‐DLBCL) and U2932 (ABC‐DLBCL) cell lines were obtained from American Type Culture Collection (Rockville, MD). Rituximab‐resistant cell lines (RL 4RH and U2932 4RH) were created as described previously [17]. Other cell lines representing ABC‐DLBCL (TMD8, HBL‐1) and GCB‐DLBCL (SUDHL‐4, SUDHL‐6, SUDHL‐10, OCI‐LY2, OCI‐LY19, BJAB, HT, DB, Karpas‐422, NUDHL‐1) were obtained from Leibniz Institute/German Collection of Microorganisms and Cell Cultures (DSMZ). All cell lines were maintained in RPMI 1640 (Life Technologies, Grand Island, NY) supplemented with 10% heat‐inactivated fetal bovine serum (Atlanta Biologicals, Norcross, GA), 5 mM HEPES, 100 U/mL penicillin, and 100 µg/mL streptomycin (Life Technologies, Grand Island, NY) at 37°C and 5% CO_2_.

Neoplastic B‐cells were isolated from pre‐treatment biopsy tissue (either lymph node or extranodal tumor tissue) obtained from patients with B‐cell NHL or Hodgkin lymphoma (HL) receiving therapy at Roswell Park Comprehensive Cancer Center (RPCCC) as previously described [18].

### 2.2 Reagents and antibodies

Pevonedistat was provided by Millennium Pharmaceuticals, Inc. (Cambridge, MA), a wholly owned subsidiary of Takeda Pharmaceutical Company Limited. Venetoclax, Selinexor, A‐1331852, and Ibrutinib were obtained by Selleck Chemicals (Houston, TX). All other drugs were provided by the RPCCC Pharmacy.

Primary mouse anti‐human antibodies raised against Bak and Bax were obtained from Sigma Chemicals (St. Louis, MO), Mcl‐1 from Santa Cruz Biotechnology (Santa Barbara, CA). Primary mouse/rabbit anti‐human antibodies raised against Bid, Bim, Noxa, Beclin‐1, p65, Bcl‐2, Bcl‐XL, Puma, PARP‐1, cleaved PARP‐1 and actin were obtained from Cell Signaling (Danvers, MA). Alkaline phosphatase (AP) or horseradish peroxidase (HRP) conjugated anti‐mouse/rabbit secondary antibodies were purchased from Jackson ImmunoResearch (West Grove, PA).

Sodium ^51^chromate (^51^Cr) (Perkin‐Elmer Life Inc., Boston MA) was used in functional assays assessing antibody‐mediated complement cytotoxicity (CMC) or antibody dependent cellular cytotoxicity (ADCC). Triton X‐100, trypan blue and histopaque‐1077 were obtained from Sigma‐Aldrich Inc. (St Louis, MO). Cell Titer‐Glo Luminescent Viability Assay reagent was purchased from Promega (Madison, WI).

### 2.3 *In vitro* effects of pevonedistat as a single agent

DLBCL cell lines (0.25 × 10^6^ cells/ml) were exposed *in vitro* to pevonedistat (0.313 to 10 µM) for 24–72 h at 37°C and 5% CO_2_. Cell viability changes were determined by measuring adenosine triphosphate (ATP) content using the Cell Titer‐Glo Luminescent Viability Assay (Promega, Madison, WI). The half‐maximal inhibitory concentration (IC_50_) of pevonedistat was calculated using the Graph Pad Prism Software Version 6.04 (Graph Pad Software, Inc., La Jolla, CA). Primary tumor cells isolated from B‐cell lymphoma patients (1 × 10^6^ cells/mL) were exposed to pevonedistat (0.5 µM) for primary lymphoma samples or vehicle control (Dimethyl sulfoxide [DMSO] 0.001%) for 24, 48 and 72 h.

### 2.4 *In vitro* effects of BCL‐2 family proteins and induction of apoptosis in DLBCL

We selected DLBCL cell lines with high (U2932 and OCI‐LY2) or low (TMD8 and SUDHL4) IC_50_ values. TMD8 and U2932 represented the ABC‐DLBCL subtype; OCI‐LY2 and SUDHL4 represented the GCB‐DLBCL subtype. Cell cycle and apoptosis threshold changes following NAE inhibition were evaluated. DLBCL cell lines were exposed *in vitro* to pevonedistat (25‐125 nM) for 48 h. Pevonedistat‐ or vehicle control‐exposed cells were harvested, washed with PBS, and fixed with ice cold 70% ethanol at 4°C for 30 min. Then, cells were incubated with 5 µg/mL RNase for 30 minutes at room temperature and stained with propidium iodide (PI, 5 µg/mL; Sigma–Aldrich, St. Louis, MO) for 1 h. Differences in the cell cycle distribution following pevonedistat exposure were determined by flow cytometry on a FACSCalibur flow cytometer (BD Biosciences; San Jose, CA). Apoptosis induction was confirmed by flow cytometry using Annexin‐V PE Cyanine7/Sytox Blue staining and by Western blotting (PARP cleavage).

### 2.5 Changes in BCL‐2 family members and key regulatory apoptotic proteins in DLBCL cell lines after exposure to pevonedistat

To study the mechanisms responsible for pevonedistat antitumor activity in DLBCL, cells were exposed to pevonedistat [U2932 (50 and 250 nM), OCI‐LY2 (250 and 1000 nM), TMD8 (25 and 50 nM), and DHL4 (100 and 500 nM)] or vehicle for 48 h. Subsequently, changes in the expression of cell cycle regulatory proteins or BCL‐2 family members were determined by Western blotting.

### 2.6 *In vitro* effects of pevonedistat on nuclear factor kappa B activity in DLBCL

To assess the effects of NAE inhibition in NF‐κB activity, DLBCL cell lines, SUDHL4, TMD8, U2932, and OCI‐LY2 (2 × 10^6^ viable cells), were exposed to pevonedistat (0.5‐5 µM) for 1 and 4 h. NF‐κB activity was determined using a nuclear translocation assay using ImageStream technology, as previously described [19]. PMA/ionomycin was added to fully activate NF‐kB as a positive control.

### 2.7 *In vitro* effects of pevonedistat when combined with chemotherapy or small molecule inhibitors in DLBCL cell lines

To investigate if pevonedistat could potentiate the antitumor activity of chemotherapy agents, U2932, OCI‐LY2, TMD8, and SUDHL4 cells (0.25 × 10^6^ cells/ml) were treated with pevonedistat (0.5‐1 µM) and/or cisplatin (1.563‐50 µM), cytarabine (1.563‐50 µM), doxorubicin (0.125–4 µM), or etoposide (1.563–50 µM) for 48 h. Cell viability was determined using the CellTiterGlo assay. Alternately, the same cell lines (0.25 × 10^6^/mL) were exposed to pevonedistat (0.977–4 µM) and/or ibrutinib (0.391–250 nM), selinexor (0.0625‐10 µM), A‐1331852 (0.0977‐6.25 µM), and venetoclax (0.1‐1000 nM) for 72 h and cell viability was assessed by PrestoBlue®. Synergistic activity between pevonedistat and other agents was evaluated using the CalcuSyn Software Version 2.11 (Biosoft, Great Shelford, Cambridge, UK).

### 2.8 ^51^Cr release assay for analyzing impact of pevonedistat exposure on rituximab‐, obinutuzumab‐, and ofatumumab‐associated CMC and ADCC

DLBCL cell lines were exposed *in vitro* to pevonedistat (0.5‐1 µM) or DMSO (0.001%) and incubated at 37°C and 5% CO_2_ for 48 h. Subsequently, 2 × 10^6^ viable cells were labeled with ^51^Cr at 37°C, 5% CO_2_ for 2 h. **^51^** Cr‐labeled DLBCL cell lines were then plated at a cell concentration of 1 × 10^5^ cells/well (CMC assay) or 1 × 10^4^ cells/well (ADCC assay). Cells were then exposed to rituximab (10 µg/mL), obinutuzumab (10 µg/mL), ofatumumab (10 µg/mL) or isotype (10 µg/mL) and human serum (CMC, 1:4 dilution) or PBMCs (ADCC, 40:1 effector: target ratio) for 6 h at 37°C and 5% CO_2_. ^51^Cr release was measured, and percentage of cell‐lysis was calculated as previously described [17].

### 2.9 *In vivo* effects of pevonedistat as a single agent or in combination with rituximab, chemotherapeutic agents, or small‐molecule inhibitors in DLBCL murine models

*In vivo* studies utilized a disseminated human lymphoma‐bearing SCID mouse xenograft model. Experiments were conducted using 6‐8‐week‐old severe combined immune‐deficient (SCID) mice (SCID C.B‐Igh‐1 b/lcrTac‐Prkdcscid /Ros), bred, and maintained at facilities certified by the American Association for Accreditation of Laboratory Animal Care (AAALAC). SCID mice were inoculated on day zero with 10 × 10^6^ TMD8 cells through tail vein injection. After 72 h, the animals were then divided into eight cohorts of 15 animals each. The first cohort (group A) was used as control without any treatment. Group B was treated with pevonedistat at 180 mg/kg/dose given subcutaneously (SQ) on days +3, +6, +10, +13, +17, and +20. Group C was treated with ibrutinib at 12.5 mg/kg/dose given via gavage daily on days +3 to +21. Group D was treated with rituximab at 10 mg/kg on days +5, +12, and +19 administered via tail vein injection (IV). Group E was treated with cytarabine at 20 mg/mouse intraperitoneally (IP) on days +3, +10, and +17. Groups F, G, and H were treated with a combination of pevonedistat SQ and either ibrutinib via gavage, or rituximab IV, or cytarabine IP, respectively, at doses mentioned above. The endpoint of the studies was survival, defined as the time to development of limb paralysis.

### 2.10 Statistics

All experiments were performed in triplicates on three separate occasions. Data were plotted and analyzed using SPSS 21.0 software. For *in vitro* and ex vivo studies, statistical differences between treatment groups and controls were determined by Student's *t*‐test. For *in vitro* studies combining pevonedistat and chemotherapy drugs, the coefficient of synergy was calculated using the CalcuSyn software. In addition, differences in survival between treatment groups were calculated using Kaplan–Meier curves. *P*‐value of less than .05 was defined as having statistical significance.

## 3 RESULTS

### 3.1 Pevonedistat induces cell death in DLBCL cell lines and in primary tumor cells

**FIGURE 1:**
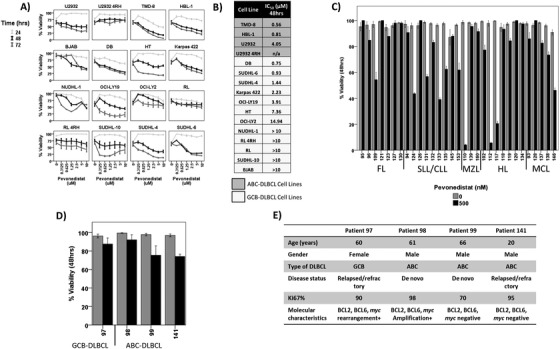
Pevonedistat induces a dose‐dependent and time‐dependent reduction in cell viability and cellular growth in DLBCL cell lines and exhibits a variable degree of activity in tumor cells derived from B‐NHL patients. (A) *In vitro* exposure of DLBCL cell lines to pevonedistat resulted in dose‐ and time‐dependent (24‐hour, 48‐hour and 72‐hour time points shown here) cell death in ABC‐DLBCL (HBL‐1, TMD8, U2932, U2932 4RH) and GCB‐DLBCL (BJAB, DB, HT, Karpas 422, NUDHL‐1, OCI‐LY2, OCI‐LY19, RL, RL4RH, SUDHL‐4, SUDHL‐6, SUDHL‐10) cells. 4RH denotes rituximab‐resistant cell lines. All cell lines were exposed to escalating doses of pevonedistat (0.313‐10 µM) for 24, 48, and 72 hours. Cell death was determined by Cell Titer Glo luminescence assay. (B) Pevonedistat IC_50_ levels at 48 h. IC_50_ was lower in the ABC‐DLBCL cell lines. (C) Ex vivo exposure of primary tumor cells derived from patients with B‐cell malignancies such as follicular lymphoma, small lymphocytic leukemia/chronic lymphocytic leukemia, marginal zone lymphoma, Hodgkin's lymphoma and mantle cell lymphoma to pevonedistat resulted in varying degrees of cell death. Tumor cells were obtained by B‐cell enrichment of fresh biopsy samples. (D) Ex vivo exposure of primary tumor cells derived from patients to pevonedistat lead to greater than 20% decrease in viability in two of three ABC‐DLBCL samples whereas no effect was noted in a GBC‐DLBCL sample. (E) Clinical and pathological characteristics from which primary tumor cells derived from DLBCL patients were obtained. Experiments were repeated three separate times and were reported as the median with standard deviation error bars (SE). Pevonedistat or vehicle control was utilized at 500 nM in patients with DLBCL. Cell death was determined by Cell Titer Glo luminescence assay. *P < .05

Exposure to pevonedistat led to a time‐ and dose‐dependent decrease in viability for all cell lines evaluated. IC_50_ concentrations varied between 0.56 µM for the most sensitive (TMD8) and 4.05 µM for the most resistant (U2932) ABC‐DLBCL cell lines, and between 0.75 µM for the most sensitive (DB) and 14.94 µM for the most resistant (OCI‐LY2) GCB‐DLBCL cell lines (Figure 1A and B) at 48 h. Rituximab‐resistant cell lines did not show dose‐dependent cell death, and IC_50_ values were in‐evaluable. A variable degree of cell death was observed in primary tumor cells isolated from patients with B‐cell lymphoma (Figure 1C), including DLBCL with patient characteristics as described (Figure 1D and 1E). Two out of four DLBCL patient samples (both ABC‐DLBCL subtype) showed cell death (Figure 1D).

### 3.2 Pevonedistat *in vitro* exposure altered the balance of several BCL‐2 family member proteins favoring the induction of apoptosis in DLBCL

**FIGURE 2:**
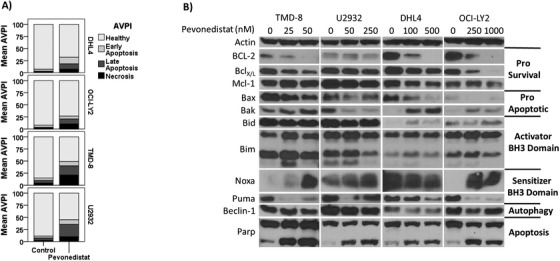
Pevonedistat *in vitro* exposure altered the balance of Bcl‐2 family member proteins favoring the induction of apoptosis. (A) DLBCL cell lines were exposed to pevonedistat (SUDHL4 100 nM, OCI‐LY2 125 nM, TMD8 25 nM, and U2932 50 nM), Sytox Blue and PE‐Cyanine7 Annexin‐V, fixed and analyzed by flow cytometry. The ABC‐DLBCL cell lines (TMD8 and U2932) were more sensitive to pevonedistat compared to the GCB‐DLBCL cell lines (SUDHL4 and OCI‐LY2). Experiments were repeated three separate times and were reported as the median. (B) *In vitro* exposure of two ABC‐DLBCL cell lines (TMD8 and U2932) and GCB‐DLBCL cell lines (SUDHL‐4 and OCI‐LY2) to pevonedistat for 48 hours altered the balance of pro‐apoptotic (Bak) and anti‐apoptotic (BCL‐2 and Bcl‐XL) Bcl‐2 family members leading to PARP cleavage. Cells were treated with Pevonedistat (IC_25_ and IC_50_, derived from Annexin PI staining) for 48 hours followed by protein extraction. Protein was separated using a 12% sodium dodecyl sulfate (SDS) PAGE gel and transferred to a polyvinylidene difluoride (PVDF) membrane. Primary rabbit anti‐human antibodies were used a 1:1000 while secondary anti‐rabbit antibodies were used at 1:10000

We selected DLBCL cell lines with high (U2932 and OCI‐LY2) or low (TMD8 and SUDHL4) IC_50_ values. TMD8 and U2932 represented the ABC‐DLBCL subtype, OCI‐LY2 and SUDHL4 represented the GCB‐DLBCL subtype. Induction of apoptosis at 48 hours was detected by flow cytometry in ABC‐ and to a lesser degree, GCB‐DLBCL cell lines (Figure 2A). Pevonedistat exposure at 48 hours induced downstream apoptotic pathway changes more effectively in ABC‐DLBCL, as evidenced by PARP cleavage (using lower drug concentrations), when compared with GCB‐DLBCL cell lines (Figure 2B). In all cell lines, pevonedistat (used at IC_50_ drug concentrations, derived from the Annexin/PI staining for apoptosis experiment) induced a dose‐dependent decrease in the expression of BCL‐2 and BCL‐XL in all cell lines and induced a dose‐dependent upregulation of the pro‐apoptotic protein, BAK in TMD‐8, DHL4 and OCI‐LY2 (Figure 2B). Downregulation of PUMA was also noted. These effects were less pronounced in theU2932 cell line.

### 3.3 Pevonedistat decreased NF‐κB activity in DLBCL cell lines

**FIGURE 3:**
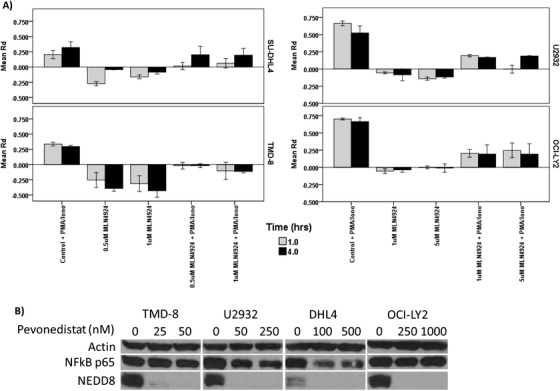
*In vitro* exposure of DLBCL cell lines to pevonedistat decreases NFκB activity. (A) A panel of DLBCL cell lines were exposed to pevonedistat (0.5‐5 µM) for 1 and 4 h. Changes in NFκB activation were analyzed using ImageStream technology. Nuclear p65 translocation into the nuclei is reported as similarity score (SS) of Dapi and p65 images. The RD metric reports the changes in SS by measuring the shift between two distributions. (B) Changes in NFκB p65 expression were analyzed by western blotting following pevonedistat *in vitro* exposure. NFκB p65 expression was decreased in U2932 and DHL‐4, but not in TMD8 and OCI‐LY2

NF‐κB activity is known to be tightly regulated by the UPS, and translocation of p65 into the nucleus leads to the increased transcription of anti‐apoptotic Bcl‐2 family members. Similar to observations with other UPS inhibitors (i.e., bortezomib), pevonedistat decreased NF‐κB activity in the cell lines tested, as demonstrated by p65 co‐localization studies (Figure 3A). NF‐κB p65 expression was decreased in U2932 and DHL‐4 by Western blotting (Figure 3B).

### 3.4 Pevonedistat displayed synergy with chemotherapeutic agents and small molecule inhibitors *in vitro* and was highly active as a single agent and in combination *in vivo*

**FIGURE 4:**
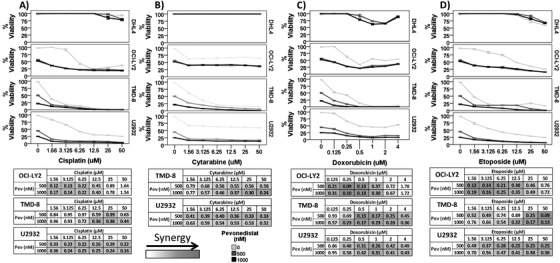
*In vitro* effects of pevonedistat on the antitumor activity of chemotherapy agents. TMD8, U2932, SUDHL‐4 and OCI‐LY2 cells were pretreated with pevonedistat for 48 h at 0.5 and 1 µM respectively. Cells were then washed and exposed to Cisplatin (1.56‐50µM) (A), Cytarabine (1.56‐50 µM) (B), Doxorubicin (0.125‐4µM) (C), or Etoposide (1.56‐50µM) (D) for 48 h. Viability was determined by Cell Titer Glo luminescence assay. Experiments were performed in triplicates. All four chemotherapeutic agents showed synergy with pevonedistat in TMD8 and U2932. Cisplatin, doxorubicin and etoposide also showed synergy with pevonedistat pretreatment in OCI‐LY2 cells, but to a lesser extent

Pre‐treatment with pevonedistat for 48 h exhibited synergistic/additive effects when combined with cisplatin, cytarabine, doxorubicin and etoposide (Figure 4A–D). The magnitude of synergy was larger in ABC‐DLBCL cell lines (TMD8 and U2932) than in GCB‐DLBCL cell lines. Strong synergy was noted between pevonedistat and the BCL‐XL inhibitor, A1331852 (Figure 5A), and between pevonedistat and venetoclax (Figure 5B), in both GCB‐ and ABC‐DLBCL cell lines. Similarly, strong synergy was noted between pevonedistat and the BTK inhibitor, ibrutinib (Figure 5C), and between pevonedistat and the selective inhibitor of nuclear export (SINE) compound, selinexor (Figure 5D) in all fourcell lines tested, suggesting a cell‐of‐origin agnostic mechanism of synergy.

FIGURE 5
*In vitro* effects on pevonedistat on the anti‐tumor activity of small molecule inhibitors. Pevonedistat demonstrated significant synergy with A‐1331852 (A), venetoclax (B), ibrutinib (C) and selinexor (D). DLBCL cells (0.25 × 10^6^/mL) were exposed to pevonedistat (0.977‐4 µM) and/or ibrutinib (0.39‐250 nM), selinexor (0.0625‐10 µM), A‐1331852 (0.0977‐6.25 µM) and venetoclax (0.1‐1000 nM) for 72 h and cell viability assessed by PrestoBlue®. Synergistic activity between pevonedistat and other agents was evaluated using the CalcuSyn Software Version 2.11 (Biosoft, Great Shelford, Cambridge, United Kingdom)

**Figure:**
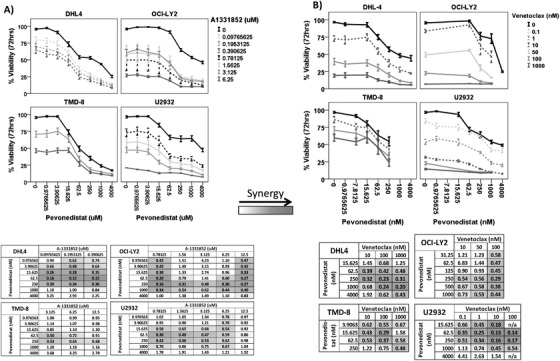

**Figure:**
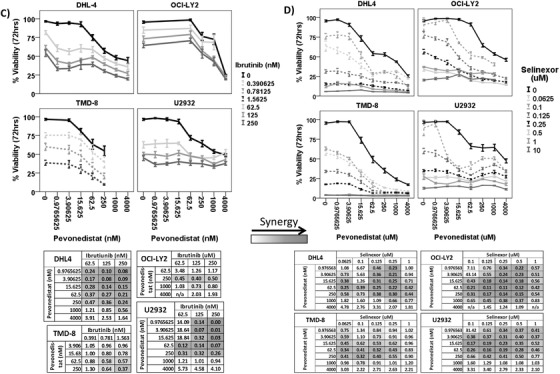

**FIGURE 6:**
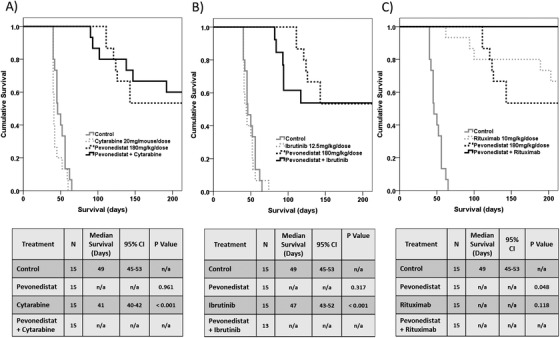
Effect of Pevonedistat on the anti‐tumor activity of cytarabine, ibrutinib and rituximab *in vivo*. *In vivo*, pevonedistat in combination with cytarabine or ibrutinib improved the median survival (not reached at the time of sacking the TMD8‐bearing SCID mice on 214th day post‐treatment) compared to cytarabine (41 days) (A) or ibrutinib (47 days) (B) monotherapy (*P* < .001). The combination of pevonedistat with rituximab did not improve survival compared to rituximab alone (C). Survival differences between groups were compared using log rank analysis. *P* values are of combination compared to single agent treatment. Experiments were repeated three separate times. (n/a = Not reached)

*In vivo*, pevonedistat alone or in combination with cytarabine or ibrutinib improved the median survival of lymphoma‐bearing SCID mice (not reached) when compared to animals treated with cytarabine (41 days) or ibrutinib (47 days) monotherapy (*P* < .001) (Figure 6A,B).

### 3.5 Effect of Pevonedistat on anti‐CD20 monoclonal antibody activity *in vitro* and *in vivo*

Previous investigators have demonstrated that ADCC is an important mechanism of action of anti‐CD20 monoclonal antibodies *in vitro* and *in vivo* [20, 21]. Pre‐incubation of DLBCL cell lines with pevonedistat for 48 h prior to rituximab exposure dampened anti‐CD20 monoclonal antibody‐mediated CMC for TMD‐8 and U2932 but enhanced CMC activity in SUDHL4 (Supporting Information Figure 1B). Results for ADCC were more consistent with pre‐incubation with pevonedistat for 48 h prior to rituximab exposure decreasing anti‐CD20 monoclonal antibody‐mediated ADCC in all 4 cell lines tested (Supporting Information Figure 1C). Additive effects were noted on apoptosis when pevonedistat was combined with rituximab in ABC‐DLBCL (Supporting Information Figure 1A). However, more importantly, additive activity was observed *in vivo* when pevonedistat was combined with rituximab in a lymphoma mouse model. TMD8‐bearing SCID mice treated with pevonedistat and rituximab resulted in a 100% long‐term disease control when compared with animals treated with pevonedistat (*P* = .048). However, there was no improvement in survival when the combination was compared to rituximab monotherapy (*P* = .118) (Figure 6C).

## 4 DISCUSSION

In DLBCL preclinical models, pevonedistat demonstrated a dose‐ and time‐dependent reduction in cell viability with varying sensitivity between ABC‐ and GCB‐DLBCL cell lines and primary tumor cells. *In vitro* exposure of DLBCL cell lines to pevonedistat resulted in changes in the expression of BCL‐2 family members suggesting a pro‐apoptotic micro‐environment, including the downregulation of BCL‐2/BCL‐XL and increased expression of BAK. Although we demonstrated a marked upregulation of NOXA levels, in contrast to other studies [22] we did not notice changes in Bim or other BH3 single‐domain proteins following pevonedistat drug exposure in the cell lines tested. Pevonedistat sensitized DLBCL cells to the cytotoxic effects of four chemotherapy drugs commonly used to treat DLBCL (i.e., cisplatin, cytarabine, doxorubicin and etoposide). Pevonedistat also exhibited cell‐of‐origin agnostic synergy with small molecules targeting the apoptotic pathway (A‐1331852, the BCL‐XL inhibitor and venetoclax, the BCL‐2 inhibitor) and the SINE compound, selinexor. Its synergy with ibrutinib was more pronounced in the ABC‐DLBCL cell lines. Significant survival benefit was noted in mouse models with pevonedistat alone or in combination with cytarabine or ibrutinib when compared with cytarabine or ibrutinib monotherapy. In contrast to what we observed in mantle cell lymphoma (MCL) pre‐clinical models [23], pevonedistat did not consistently improve anti‐tumor activity of rituximab in DLBCL.

Pevonedistat induces a pro‐apoptotic milieu and downregulates NF‐κB activity, which makes it an attractive partner for drug combinations. Prior studies have demonstrated that in CLL and DLBCL, pevonedistat restored sensitivity to death receptor‐mediated apoptosis through the mitochondrial pathway [24]. In CLL, it also induced DNA damage and checkpoint activation by deregulation of Cdt1, a DNA replication licensing factor, and cell cycle inhibitors p21 and p27 [25]. It exhibited synergy with alkylating agents such as bendamustine and chlorambucil [25]. In our study, while significant *in vitro* synergy was noted with a variety of chemotherapeutic agents and small molecule inhibitors, we could not replicate similar results *in vivo* as our model incorporating TMD8 ABC‐DLBCL cell lines into SCID mice was quite sensitive to pevonedistat, even as a single agent. Further experiments at varying doses will be needed to demonstrate synergy between these agents in *in vivo* mouse models. It is also interesting to note the variability of responses between different cell lines. The next line of investigation will be to identify factors causing primary resistance to pevonedistat in cell lines and translate that data to identify patients who stand to benefit the most from this therapy and find strategies to overcome both primary and acquired resistance.

The molecular events observed in lymphoma or CLL pre‐clinical models using pevonedistat are similar to what has been demonstrated with reversible (bortezomib or ixazomib) or irreversible (carfilzomib) proteasome inhibitors [26, 27]. In DLBCL, the anti‐tumor activity of proteasome inhibitors appears to be limited to the ABC‐DLBCL subtype [7]. Clinically, the activity of proteasome inhibitors in lymphoid malignancies has been limited by toxicities. Early clinical trials evaluating bortezomib in combination with systemic chemotherapy in patients with relapsed DLBCL demonstrated anti‐tumor activity in ABC‐DLBCL [7]. In contrast, a large randomized clinical trial comparing rituximab and CHOP chemotherapy vs. rituximab, cyclophosphamide, doxorubicin, bortezomib, and prednisone in previously untreated ABC‐DLBCL failed to meet its primary endpoint [28]. The phase 3 REMoDL‐B study also demonstrated no differences in outcomes with the addition of bortezomib to R‐CHOP in patients with either ABC‐ or GCB‐DLBCL [29]. While studies evaluating the addition of carfilzomib to various chemotherapy backbones in DLBCL are ongoing [8, 30], there is a clinical need to develop novel and less toxic agents targeting the UPS system such as pevonedistat. Preclinical data, including our current work, support the development of NAE inhibitors in DLBCL.

Pevonedistat has been studied as a single agent in a phase 1 trial in patients with relapsed/refractory multiple myeloma and lymphoma [31]. The drug was well tolerated with modest activity; dose‐limiting toxicities included febrile neutropenia, transaminase elevations, muscle cramps, and thrombocytopenia. It is now being studied in a host of solid and hematological malignancies in various combinations. Based on the preclinical synergy between pevonedistat and ibrutinib in CLL, MCL, and now in DLBCL [22, 32], a phase 1 trial combining the two drugs in patients with relapsed/refractory CLL or NHL including ABC‐DLBCL (NCT03479268), is currently ongoing and actively recruiting patients at our institution.

In the future, combinations of pevonedistat and second generation BTK inhibitors such as acalabrutinib, zanubrutinib and LOXO‐305 merit investigation due to their enhanced safety profile. While pevonedistat did demonstrate *in vitro* synergy with small molecule inhibitors such as the SINE compound, selinexor, BCL‐XL inhibitor, A‐1331852 and BCL2 inhibitor, venetoclax, the mechanisms of synergy between these agents have to be further elucidated before these combinations can be studied in patients. While pevonedistat is active in many disease settings, relapsed DLBCL is a promising area of interest as adding pevonedistat to cytarabine‐containing salvage regimens may lead to higher response rates without compromising safety.

## FUNDING INFORMATION

This work was supported, in part, by grants from the National Cancer Institute (5RO1CA136907‐02), the Eugene and Connie Corasanti Lymphoma Research Fund, the Czech Ministry of Health grant IGA‐MZ NT13201‐4/2012 and institutional research grants PRVOUK‐27/LF1/1 and PRVOUK P24/LF1/3.

## AUTHOR CONTRIBUTIONS

P.T., S.K., C.M., S.B., and J.G. performed research; P.T., J.G., F.J.H.I. designed the research study; P.T., S.K., C.M., J.G., and F.J.H.I. analyzed the data; P.T., S.K., C.M. J.G., S.S., M.B, and F.J.H.I., wrote and edited the paper. All authors give their approval for the final manuscript.

## CONFLICT OF INTEREST

F.J.H.I. has served on advisory boards for Millennium Pharmaceuticals, Pharmacyclics, Celgene, Amgen, Genentech, and Seattle Genetics. The remaining authors declare no competing interest.

## Supporting information


**Figure S1: Effect of pevonedistat on activity of anti‐CD20 monoclonal antibodies. (A)** Pevonedistat at (SUDHL4 100 nM, OCI‐LY2 125 nM, TMD8 25 nM, and U2932 50 nM) had an additive effect on apoptosis induced by rituximab in ABC‐DLBCL cell lines. Pre‐incubation of DLBCL cell lines for 48 hours with pevonedistat decreased rituximab, ofatumumab or obinutuzumab‐associated cell mediated cytotoxicity (CMC) **(B)** and antibody dependent cellular cytotoxicity (ADCC) **(C)** *in vitro*. Both, ABC‐DLBCL and GCB‐cell lines were evaluated. DLBCL cell lines were exposed *in vitro* to pevonedistat (0.5‐1µM) or DMSO (0.001%) and incubated at 37°C and 5% CO2 for 48 hours. Subsequently, 2 × 10^6^ viable cells were labeled with ^51^Cr at 37°C, 5% CO2 for 2 hours. ^51^Cr‐labeled DLBCL cell lines were then plated at a cell concentration of 1 × 10^5^ cells/well (CMC assay) or 1 × 10^4^ cells/well (ADCC assay). Cells were then exposed to rituximab (10 µg/ml), obinutuzumab (10 µg/ml), or isotype (10 µg/ml) and human serum (CMC, 1:4 dilution) or PBMCs (ADCC, 40:1 effector: target ratio) for six hours at 37°C and 5% CO2. ^51^Cr release was measured and percentage of cell‐lysis was calculated as previously described. Each experiment was done in triplicate.
