# Supplementary figures and images for: Pevonedistat, a NEDD8‐activating enzyme inhibitor, induces apoptosis and augments efficacy of chemotherapy and small molecule inhibitors in pre‐clinical models of diffuse large B‐cell lymphoma
Source: EJHaem. 2020 Apr 9;1(1):122–32. doi: 10.1002/jha2.2 (PMC7566777; doi:10.1002/jha2.2)

## Slide 1
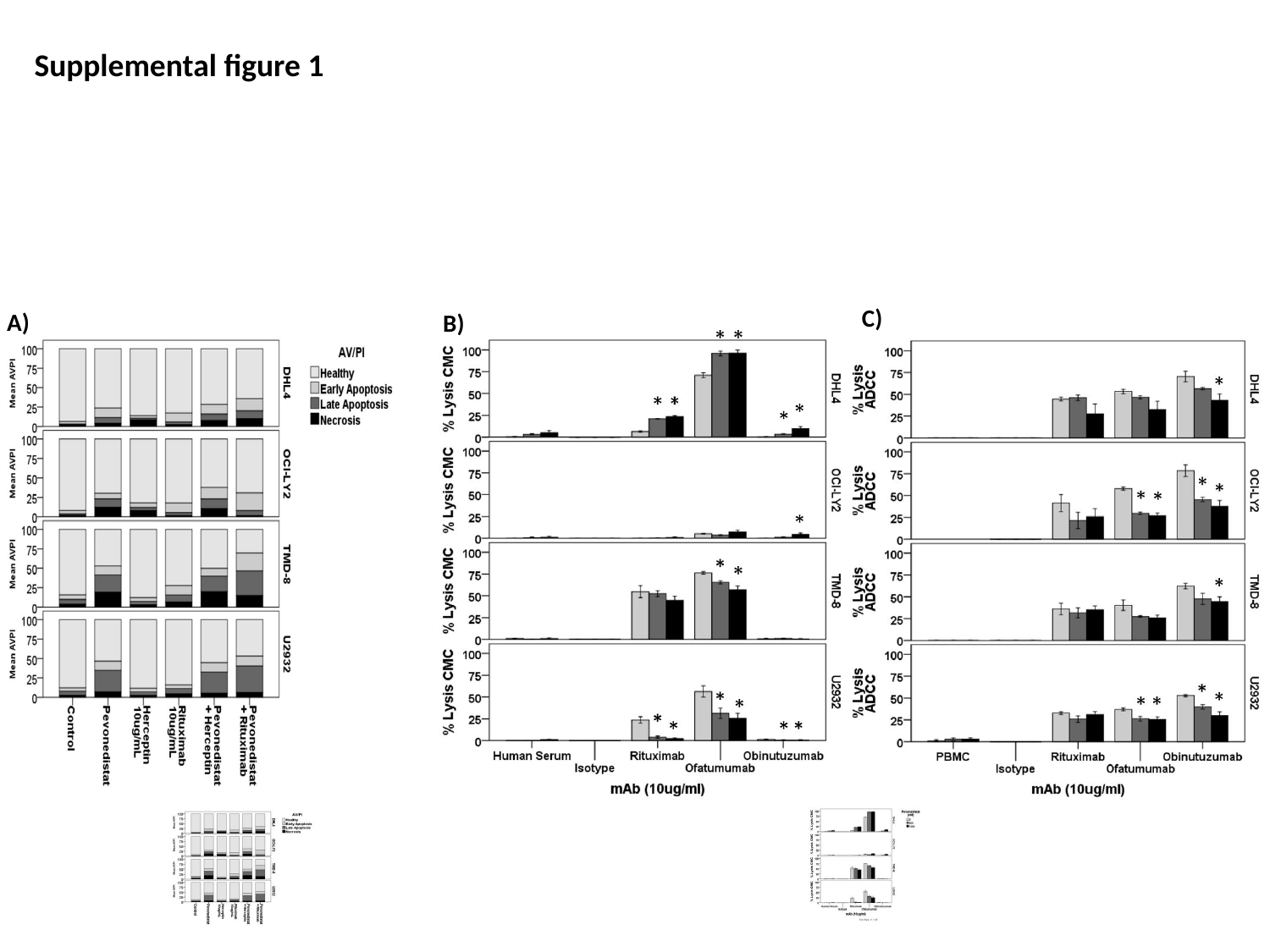

Supplemental figure 1
C)
A)
B)

Supplement: Supplementary file 1 — Figure S1: Effect of pevonedistat on activity of anti‐CD20 monoclonal antibodies. (A) Pevonedistat at (SUDHL4 100 nM, OCI‐LY2 125 nM, TMD8 25 nM, and U2932 50 nM) had an additive effect on apoptosis induced by rituximab in ABC‐DLBCL cell lines. Pre‐incubation of DLBCL cell lines for 48 hours with pevonedistat decreased rituximab, ofatumumab or obinutuzumab‐associated cell mediated cytotoxicity (CMC) (B) and antibody dependent cellular cytotoxicity (ADCC) (C) in vitro. Both, ABC‐DLBCL and GCB‐cell lines were evaluated. DLBCL cell lines were exposed in vitro to pevonedistat (0.5‐1µM) or DMSO (0.001%) and incubated at 37°C and 5% CO2 for 48 hours. Subsequently, 2 × 10^6^ viable cells were labeled with 51Cr at 37°C, 5% CO2 for 2 hours. 51Cr‐labeled DLBCL cell lines were then plated at a cell concentration of 1 × 10^5^ cells/well (CMC assay) or 1 × 10^4^ cells/well (ADCC assay). Cells were then exposed to rituximab (10 µg/ml), obinutuzumab (10 µg/ml), or isotype (10 µg/ml) and human serum (CMC, 1:4 dilution) or PBMCs (ADCC, 40:1 effector: target ratio) for six hours at 37°C and 5% CO2. 51Cr release was measured and percentage of cell‐lysis was calculated as previously described. Each experiment was done in triplicate. [file JHA2-1-122-s001.pptx]
